# A Rare Presentation of Diffuse Large B-Cell Lymphoma as a Gastric Outlet Obstruction With Complete Resolution After Chemotherapy

**DOI:** 10.7759/cureus.96515

**Published:** 2025-11-10

**Authors:** Antony J Arumairaj, Yamama Al-Khazraji, Sajan Acharya, Aleksandra Mamorska-Dyga, Andrea Popescu-Martinez

**Affiliations:** 1 Internal Medicine/Section of Pulmonary Critical Care Medicine, Wake Forest University School of Medicine, Winston-Salem, USA; 2 Internal Medicine, Lutheran Main Hospital, Fort Wayne, USA; 3 Internal Medicine, Woodhull Medical Center, New York, USA; 4 Internal Medicine/Division of Hematology Oncology, Montefiore Nyack Hospital, Nyack, USA; 5 Internal Medicine/Division of Hematology Oncology, New York University (NYU) Langone Health, New York, USA

**Keywords:** chemotherapy, diffuse large b-cell lymphoma, gastric outlet obstruction, non-hodgkin lymphoma, remission

## Abstract

Diffuse large B-cell lymphoma (DLBCL) is the most common type of non-Hodgkin lymphoma (NHL), accounting for approximately one-third of all cases. We report a case of DLBCL presenting as gastric outlet obstruction (GOO) with complete resolution of obstruction after chemotherapy without any surgical intervention. A 58-year-old man presented with an inability to tolerate oral intake, bilious vomiting, abdominal discomfort, and unintentional weight loss. Computed tomography (CT) of the abdomen and pelvis revealed large infiltrating retroperitoneal and mesenteric masses with extensive adenopathy. Conservative management of GOO was initiated, including gastric decompression using a nasogastric tube and intravenous fluid hydration. A para-aortic lymph node biopsy confirmed the diagnosis of DLBCL. Following the first cycle of chemotherapy, the patient showed remarkable improvement. A repeat CT scan of the abdomen demonstrated complete resolution of GOO. Gastric outlet obstruction secondary to DLBCL is a rare complication. Complete resolution of GOO with radiological evidence following chemotherapy alone, without surgical intervention, is rarely reported.

## Introduction

Diffuse large B-cell lymphoma (DLBCL) is the most common type of non-Hodgkin lymphoma (NHL), accounting for approximately 25% to 30% of all NHL cases [1]. DLBCL is an aggressive, fast-growing cancer that affects B-lymphocytes. The disease is more common in males than females and typically affects patients in their sixth decade of life. The most common symptom of DLBCL is one or more painless swellings, usually in the neck, armpit, or groin [2]. Other symptoms may include fever, night sweats, weight loss, abdominal pain, bone pain, shortness of breath, dysphagia, and reddened patches or lumps on the skin. The standard treatment for DLBCL is a combination of chemotherapy and the monoclonal antibody rituximab, which can lead to disease remission in many patients [3]. The most widely used treatment regimen is rituximab, cyclophosphamide, doxorubicin, vincristine, and prednisone (R-CHOP regimen), typically administered in 21-day cycles for an average of six cycles [3]. In cases where the disease no longer responds to chemotherapy, other treatment options may include immunotherapy such as chimeric antigen receptor (CAR) T-cell therapy, monoclonal antibodies, or targeted therapy drugs [4]. Despite its aggressive nature, DLBCL is potentially curable, with survival rates varying depending on the stage of the disease and other prognostic factors. Without treatment, the median survival for patients with DLBCL is less than one year [5]. However, survival rates have improved significantly in recent decades due to advances in therapy, particularly the addition of rituximab to chemotherapy regimens [6]. Gastric outlet obstruction (GOO) is a relatively uncommon complication of DLBCL. We present a rare case of DLBCL manifesting with GOO. The patient’s successful treatment with R-CHOP and resolution of the obstruction after the first cycle of chemotherapy demonstrate the clinical effectiveness of this regimen in managing such complicated presentations. Although complications during R-CHOP treatment can be challenging, overall outcomes are generally favorable, underscoring the importance of this therapeutic approach for patients with DLBCL and gastric involvement [7].

## Case presentation

**Figure 1:**
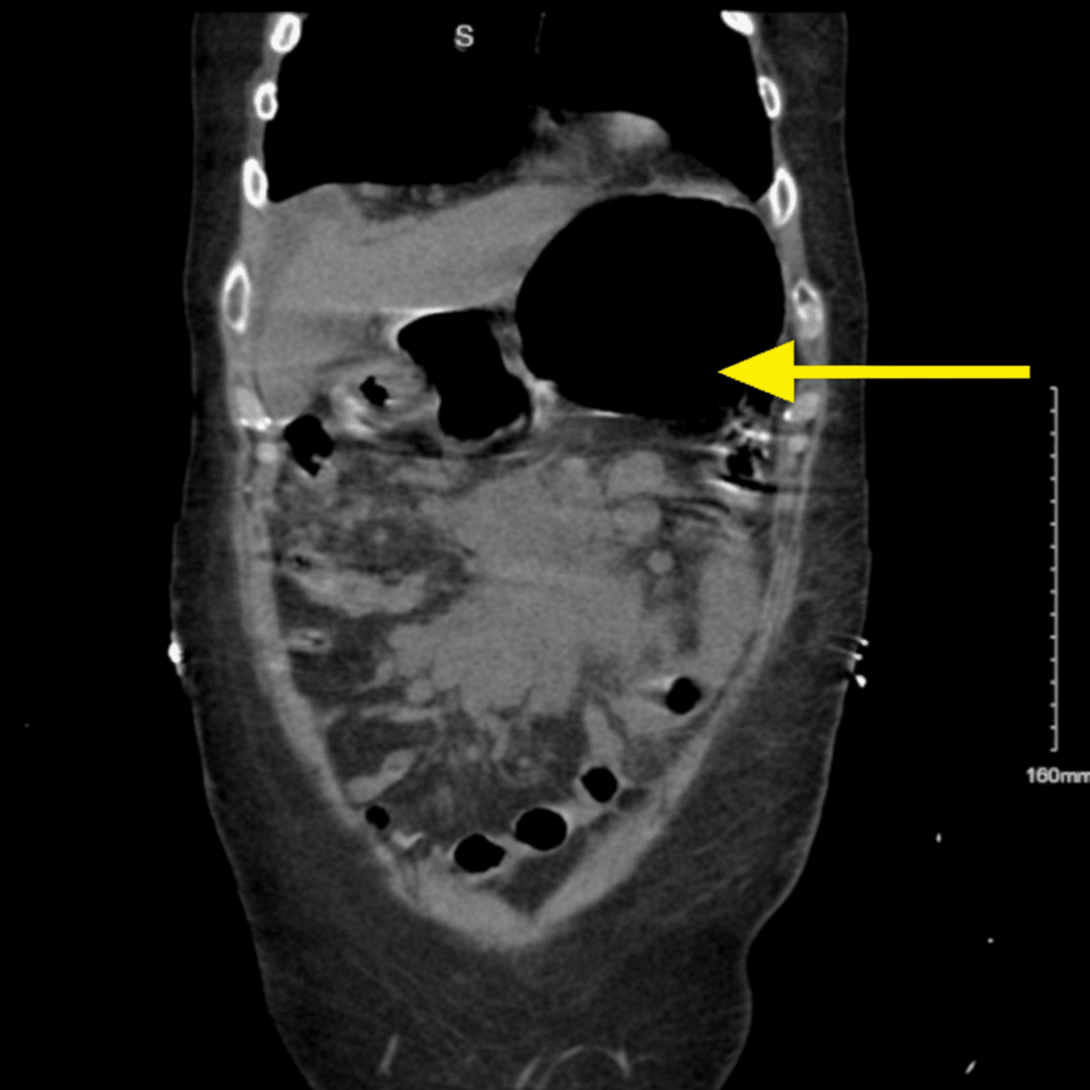
Coronal section of CT abdomen and pelvis showing the gastric dilatation secondary to the gastric outlet obstruction The yellow arrow indicates the dilated stomach from gastric outlet obstruction.

A 58-year-old man presented to the emergency department with an inability to tolerate oral intake, bilious vomiting, and abdominal discomfort for five days, along with unintentional weight loss for two months. On evaluation, the patient had a heart rate of 106 beats per minute and blood pressure of 124/75 mmHg. Examination revealed a chronically ill-looking man with mild temporal wasting and axillary lymphadenopathy. Abdominal examination showed a palpable mass in the left upper quadrant of the abdomen. Laboratory investigations revealed hemoglobin of 7.4 g/dL, differential count showing neutrophils 67.4%, lymphocytes 22.1%, monocytes 4.8%, eosinophils 4.6%, hyponatremia with serum sodium of 128 mEq/L, hypochloremia with serum chloride of 82 mEq/L, and elevated lactate dehydrogenase (LDH) of 354 units per liter (U/L). Computed tomography (CT) of the abdomen and pelvis demonstrated large infiltrating retroperitoneal and mesenteric masses with extensive lymphadenopathy. The CT also showed a fluid-filled stomach and descending part of the duodenum, consistent with gastric outlet obstruction, as shown in Figure 1. Conservative management of gastric outlet obstruction was initiated. A nasogastric tube was inserted for gastric decompression, and the patient was started on intravenous fluid hydration.

**Figure 2:**
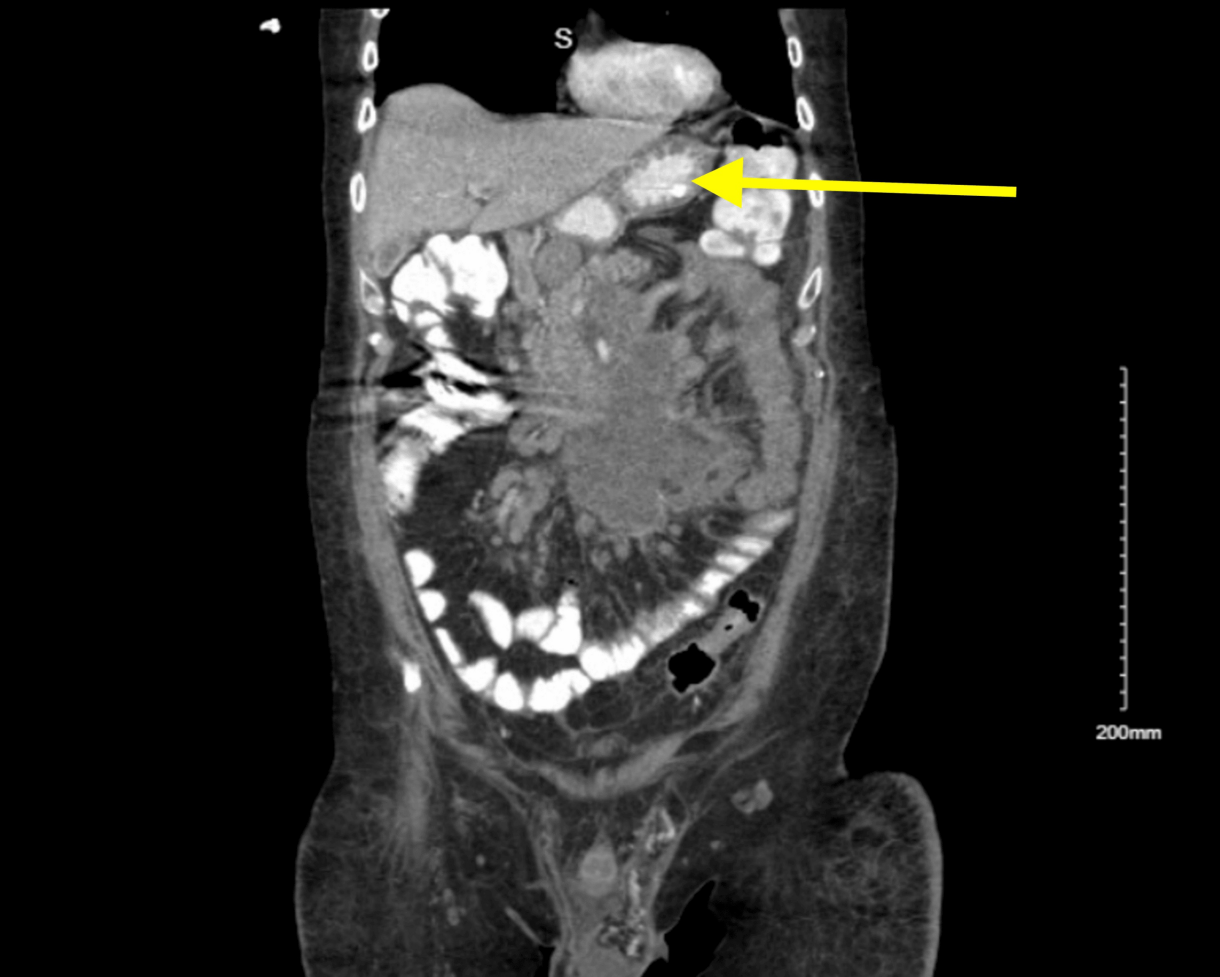
Coronal section of CT abdomen and pelvis The yellow arrow shows the normal-sized stomach with complete resolution of the gastric dilatation and gastric outlet obstruction after chemotherapy.

The para-aortic lymph node was biopsied under CT guidance, and DLBCL of germinal center B-cell origin was diagnosed. The patient was started on a treatment regimen with rituximab, cyclophosphamide, doxorubicin, vincristine, and prednisone (R-CHOP regimen) for stage IV DLBCL with curative intent. Following the first cycle of R-CHOP, the patient experienced significant improvement. A repeat CT of the abdomen and pelvis showed complete resolution of the GOO, as shown in Figure 2. The patient was gradually transitioned to oral feeds and made a complete recovery from the GOO. He was discharged to a subacute rehabilitation facility. He continued follow-up with oncology and continued his chemotherapy as an outpatient. The patient encountered multiple complications, including sepsis, febrile neutropenia, multilobar pneumonia, fungemia, and supraventricular tachycardia during his chemotherapy course, necessitating intensive care unit admissions, all of which were managed successfully. The patient completed six cycles of the R-CHOP regimen over a period of eight months. After completion of the R-CHOP regimen, a positron emission tomography (PET) scan revealed markedly decreased retroperitoneal and mesenteric lymphadenopathy with no fluorodeoxyglucose (FDG) avidity. His last bone marrow biopsy showed no involvement with lymphoma. The patient achieved complete remission from DLBCL.

## Discussion

The major causes of GOO are peptic ulcer disease, gastric polyps, ingestion of caustics, pyloric stenosis, congenital duodenal webs, gallstone obstruction, pancreatic pseudocysts, and bezoars [8]. Pancreatic cancer is the most common malignancy causing GOO, which may occur in 10-20% of patients with pancreatic carcinoma [9]. Other tumors that may obstruct the gastric outlet include ampullary cancer, duodenal cancer, cholangiocarcinoma, gastric cancer, and metastases to the gastric outlet [10]. GOO as a result of DLBCL is a rare complication and a rare presentation. The standard treatment for DLBCL is R-CHOP, a combination of chemotherapy and targeted therapy that includes rituximab, cyclophosphamide, doxorubicin, vincristine, and prednisone [11]. Our patient was diagnosed with stage IV DLBCL of germinal center type. He was treated with R-CHOP, which resulted in significant improvement and complete resolution of his gastric outlet obstruction (GOO).

A study on complications and outcomes in DLBCL patients with gastric lesions found that overall, 86% of the cohort achieved complete remission. However, gastric complications occurred in 8% of the cohort, and gastric stenosis occurred in 3% of the cohort during the first two to four cycles of R-CHOP therapy, suggesting that GOO could result from healing, scarring, and fibrosis at the site of the initial tumor [7]. Patients with gastric complications had a lower R-CHOP completion rate and a lower complete remission rate than those without complications [7]. In another case study, a patient with obstructing duodenal DLBCL and peritoneal lymphomatosis also received R-CHOP treatment, resulting in an exceptional response [12]. These clinical studies and case reviews demonstrate that R-CHOP is an effective treatment for DLBCL, including cases with gastric involvement and complications such as GOO.

Our patient had multiple complications during R-CHOP treatment, including sepsis, supraventricular tachycardia, febrile neutropenia, multilobar pneumonia, and fungemia. Some of these complications, such as infections, are common side effects of R-CHOP treatment due to immunosuppression [13,14]. Another study reported that 22.6% of patients with primary gastrointestinal DLBCL experienced complications such as bleeding, obstruction, and perforation after chemotherapy [15]. These findings indicate that complications during R-CHOP treatment are not uncommon. In our case, the patient underwent six cycles of R-CHOP over eight months, resulting in a significant reduction of retroperitoneal and mesenteric lymphadenopathy, with no lymphoma involvement noted in the last bone marrow biopsy. This outcome is consistent with the general prognosis for patients with DLBCL treated with R-CHOP, where 60-70% of patients remain progression-free after five years [16]. Since DLBCL is a highly chemosensitive tumor, patients usually have a remarkable recovery with R-CHOP chemotherapy. However, surgical intervention is essential in the management of complications such as perforation and severe bleeding [17].

## Conclusions

Diffuse large B-cell lymphoma presenting as a retroperitoneal and mesenteric mass resulting in gastric outlet obstruction is a rare complication and a rare presentation. This condition can pose a significant diagnostic challenge to physicians. However, a high index of suspicion led to the biopsy of the para-aortic lymph node, which confirmed the diagnosis of DLBCL. Prompt initiation of R-CHOP chemotherapy resulted in successful resolution of GOO secondary to DLBCL, eliminating the need for surgical intervention.
